# Maasai mother's knowledge on complementary feeding practices and nutritional status of children aged 6–24 months in Monduli District, Arusha, Tanzania: A case study of Naitolia village

**DOI:** 10.1002/fsn3.3492

**Published:** 2023-06-13

**Authors:** Jovin Binamungu, Sharadhuli I. Kimera, Beatha Mkojera

**Affiliations:** ^1^ Department of Veterinary Medicine and Public Health Sokoine University of Agriculture Morogoro Tanzania; ^2^ Department of Food Science and Agro‐Processing Sokoine University of Agriculture Morogoro Tanzania

**Keywords:** complementary feeding, dietary diversification, Maasai mothers, minimum acceptable diet, nutritional status

## Abstract

Despite Tanzania's achievement in reducing childhood problems, undernutrition is still a problem. Little is known about how mothers' knowledge on complementary feeding practice affects their children nutritional status. Therefore, the study determined how nutritional status of Maasai children aged 6–24 months is related to their mothers or caregivers' knowledge on complementary feeding. A semistructured questionnaire was used in analytical cross‐sectional study including 286 Maasai mothers and their 6–24‐month‐old children. A convenient and snowball sampling were employed in choosing households and mothers. Using SPSS version 20 and ENA for SMART software, demographic variables, mother's complementary feeding knowledge and practices, and anthropometric data were examined. Respondents were mostly young female aged 29 ± 9.5 years, married (89.2%), housewives (88.8%), with no formal education (39.1%). Maasai mothers (51.1%) introduced complementary foods at 4 months. Of all children, 75.2% did not attain minimum acceptable diet, whereas 66.1% and 57.3% did not meet minimum number of meals per day and recommended variety of foods, respectively. Based on complementary feeding practices, underweight was associated with timely introduction of complementary foods (*p* = .000), minimum dietary diversity (*p* = .001), and minimum acceptable diet (*p* = .001). Stunting was associated with minimum acceptable diet (*p* = .0027). Regarding mother's knowledge, underweight was associated with breastfeeding duration (*p* = .000) and meals adequacy (*p* = .014). Wasting was associated with breastfeeding duration (*p* = .027). Maasai mothers' weaning practices were unsatisfactory and children's nutritional status was poor. Children's nutritional status was significantly associated with mother's understanding on complementary feeding, which was only somewhat adequate.

## Graphical abstract

Despite Tanzania's achievement in reducing childhood problems, undernutrition is still a problem. Little is known about how mothers' knowledge on complementary feeding practices affects nutritional status of their children. Therefore, the study determined how nutritional status of Maasai children aged 6–24 months is related to their mothers or caregivers' knowledge on complementary feeding.

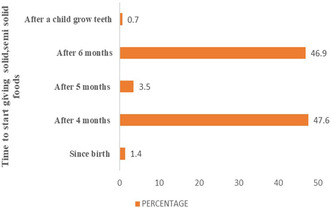

## 1 INTRODUCTION

The major foundation for childhood development is infant and young child feeding practices (Binns et al., 2017). Unfavorable factors such as inappropriate breastfeeding and infants feeding practices have adverse effects on the health and nutritional status of the children, and upon turning 6 months old, an infant's nutritional and energy needs start to outpace that is obtained from breast milk, therefore a need for additional foods (WHO, 2016); therefore, for child to have optimal growth and development, proper feeding practices are essential (Black et al., 2008). Poor breastfeeding patterns, low nutrient density, and poor quality of complementary foods account for the nutrient deficiencies and illness in children resulting in malnutrition at early age (Yimer, 2018). Malnutrition has propounding impact on child's growth and development as it can result in permanent stunting, which is linked to slow cognitive growth, lower academic and professional performance, as well as low economic output (Prado & Dewey, 2014). WHO recommended infants and young children aged 6–24 months continued breastfeeding, feeding semisolids or solid foods according to the age of the child, and feeding variety of foods such as cereals, fruits, and vegetables. In low‐ and lower middle‐income countries only one in every six children attain a minimum acceptable diet and around 25% of infants between the ages of 0 and 5 months are exclusively breastfed (UNICEF, 2018). Despite the fact that breastfeeding is the norm in Tanzania, there are many common practices including prelacteal feeding, a brief period of exclusive breastfeeding, improper meal preparation, incorrect timing for initiating complementing foods to infants, infrequent mealtimes, and foods with low‐energy and ‐nutrient contents (Muhimbula & Issa‐Zacharia, 2010; Safari et al., 2013). One third (34%) of infants and young children in Tanzania are stunted, 5% are wasted, and 14% are underweight, according to the Tanzania MoHCDGEC and Tanzania Demographic Health Survey (2015–2016) study. These levels are unacceptably high (TDHS 2015–2016). However, there are geographical variances; for example, Dar es Salaam has the lowest frequency of stunting at 15% and Rukwa has the most at 56%, while Arusha region has the highest at 36% (TDHS 2015–2016).

Inadequate information regarding complementary feeding practices among mothers is the one among underlying causes of undernutrition among children. Poor infants and young child feeding practices as well as unacceptable levels of malnutrition have been well documented in Tanzania (Kulwa et al., 2015; Safari et al., 2013). While mother's or caregivers' knowledge on the quality, quantity, frequency, and consistency have not been adequately examined especially for Maasai communities, compared to children from other ethnic groups, Maasai children have a 2–3 times higher risk of having stunted growth and wasting (Lawson, 2014). Therefore, a need for adoption of methods to improve weaning and nutritional status of Maasai children requires an understanding of the existing knowledge that influence infants and young children feeding practices. The purpose of this study was to determine how nutritional status of Maasai children aged 6–24 months in Monduli District is related to their mothers' knowledge on complementary feeding practices.

## 2 MATERIALS AND METHODS

### 2.1 Study area

The study was conducted in Monduli District which is located in the northeastern part of the United Republic of Tanzania. It has an overall area of about 6419 km^2^. Monduli District is divided into three divisions (Manyara, Makuyuni, and Kisongo) consisting of 11 wards and 39 villages. The Maasai are the popular ethic group, accounting for over 40% of the total population. According to the national census, the district population was 158,929 in 2012. The selection of the study area considered the convenience of finding Maasai people, and also the area falls under the project “The Economics of Ecosystem and Biodiversity for Agriculture and Food Tanzania Partnership Program.” For this case, Naitolia village where more than 1800 people lives in, which comprises subvillages Ormang'wai and Engusero (TPP, 2020). The Maasai, Waarusha, Iraqw, and Barabaig are the main ethnic groups in these villages. Over 178 km^2^, there are households, and the majority (79%) make a living by raising poultry, goats, and cattle.

### 2.2 Study design

The study was conducted between February and May 2022. A cross‐sectional analytical design was employed in the study to examine the link between Maasai mother's knowledge on complementary feeding and the nutritional status of their children aged 6–24 months. The dependent variables were complementary feeding and nutritional status of children (underweight, wasting, and stunting), while independent variables were Maasai mother's knowledge on complementary feeding and complementary feeding practices.

### 2.3 Population and sample size

The target population was Maasai mothers or caregivers and their children aged 6–24 months living in the selected hamlets of Naitolia village (Ormang'wai and Engusero). The sample size was calculated by using Yamane's formula for an estimated population of 1000 mothers and their respective children (Yamane, 1967).

$$n=N/\left(1+Ne^{2}\right),$$

where, *n* = desired sample size, *N* = estimated proportion of target population with the measured features (mothers with their children aged 6–24 months), *e* = required level of precision (0.05).

So,

$$n\;=\;100/\left(1+\left(1000\right)\times \;0.05^{2}\right)$$

### 2.4 Sampling method

In view of Monduli District, a purposive sampling technique was used to select the Makuyuni ward and Naitolia village in the study, then a convenient and snowball sampling technique was used to select the households (BOMA) with mothers and their children aged 6–24 months. The study involved inclusion and exclusion parameters; only Maasai mothers of children aged 6–24 months in Naitolia village and willing to take part in the study were considered inclusive. While Maasai mothers whose children were physically deformed and sick based on maternal report and clinic cards were not involved in the study.

### 2.5 Data collection

A semistructured questionnaire was used to collect information from Maasai mothers or caregivers of children aged 6–24 months in a household setting. It included inquiries on socioeconomic and demographic features of mothers and their children, complementary feeding practices, and mother's knowledge on complementary feeding practices and child's anthropometric parameters. Three categories were used to categorize the knowledge score: low for those with a score between 1 and 10, average for those between 11 and 15, and high for those between 16 and 20. According to WHO standards, a 24‐h dietary recall was used to measure the complementary feeding practices. The child's weight and length were measured by using weight scale and a recumbent board scale, the mean was determined with a margin of error of 0.01 kg and 0.1 cm for weight and length, respectively. The survey was conducted at the respondent's home to allow the interviewer to make opportunistic observation on feeding practices such as interaction of the mothers and the child during meal time, assess the food preparation, general cooking practices, and hygiene. To understand Maasai mother's feeding practice and opinions regarding child‐feeding practices, 286 mothers were selected randomly and each was observed during a single feeding episode.

### 2.6 Data analysis and interpretation

Data were cleaned, coded, and entered into the computer using version 20 of the Statistical Package for Social Sciences and Software for Emergency Nutrition Assessment for Standardized Monitoring and Assessment of Relief and Transitions (ENA for SMART) to analyze nutritional status of children, which was subsequently exported into SPSS for further analysis. In accordance with WHO (2006), children whose weight for age is below −2, *Z* scores were considered underweight, while those who are below −3 SD of the *Z* score were considered severely underweight and those who are below the −2, *Z* scores were considered short for their age (stunted) and are chronically malnourished, while those whose weight for height is below −2, *Z* scores were considered thin or wasted. The socioeconomic and demographic data and the mother's knowledge and practice regarding complementary feeding were described using frequency, mean, standard deviation, and percentage by using SPSS software. The relationship between variables and predictors of nutrition status was examined using the chi‐square and the logistic regression test, respectively.

### 2.7 Ethical considerations

The Research Ethical Committee of the Sokoine University of Agriculture granted ethical approval for the conduct of this study. The relevant district, ward, and village officials were contacted for approval to conduct the study. Mothers or caregivers were asked for their consent before they could take part in the study's activities.

## 3 RESULTS

### 3.1 Demographic characteristics of children

In this study, 286 children were involved, with females constituting 59.46% and males constituting 40%. More than half (55.6%) were born in health facilities. The average age of the children was 14.5 (6.1) months and 38.5% of them were firstborns, placing them in the 13–24 months age range (Table 1).

**TABLE 1:** Characteristics of children's population (*N* = 286).

Variable	Response	Frequency (*n*)	%
Sex	Male	116	40.6
	Female	170	59.4
Age (months)			
Age, mean (SD)	6–12	140	49
	13–23	146	51
Birth order of the children	1 Birth	110	38.5
	2 Births	53	18.5
	3 Births	51	17.8
	≥4 Births	72	25.2
Place delivered	Health facility	159	55.6
	Home	127	44.4

### 3.2 Maasai mothers/caregiver sociodemographic variables

At the households, 286 Maasai mothers in total were surveyed. The mean age of all mothers was 29 years, with the youngest mother being 16 and the oldest being 50. Of the mothers, 89.2% were married. Nevertheless, Maasai mothers had low level of education; less than half (39.5%) had never gone to school, while only 0.7% attained high college education, a significant portion (30.8%) only have a primary school education. Only 0.7% of Maasai mothers were formally employed; 88.8% of them were stay‐at‐home mothers; 10.5% of them worked as food vendors (Table 2).

**TABLE 2:** Maasai mothers/caregiver sociodemographic variables (*N* = 286).

Variable	Response	Frequency (*n*)	%
Age (years)	16–20	28	9.8
	21–30	177	61.9
	31–50	81	28.3
Marital status	Married	255	89.2
	Single	6	2.1
	Separated	7	2.4
	Widow	18	6.3
Mother's education level	Nonformal	113	39.5
	Primary school	110	38.8
	Secondary school	60	21.4
	Vocational/college	2	0.7
Occupation	Housewife	254	88.8
	Petty trade/vendors	30	10.5
	Waged labor	2	0.7

### 3.3 Maasai mothers' complementary feeding knowledge

The study's knowledge tests were constructed on WHO standards complementary feeding for breastfed children (WHO, 2003). About 50.3% of Maasai mothers stated that around the age of 1, children can eat out of the family pot. About 72.4% of Maasai mothers believed that bottle feeding was preferable for children who had refused to be breastfed. And 5.6% of the Maasai mothers reported that sick children and those recovering from illness should not be fed on diluted porridge or fruit juices; 76.9% said that the mother should assist her child to eat up to the age of 24 months. One to two tablespoons of food, according to 84.3% of Maasai mothers, is sufficient for a kid who is 12 months old. A higher number (96.5%) of the Maasai mothers knew that fruits and vegetables should be a child's diet during complementary feeding (Table 3). Less than half (46.9%) of the Maasai mothers were aware that complementary foods should be provided to children at 6 months in order to ensure optimal intakes, while more than half (51.1%) of the Maasai mothers thought that complementary feeding should begin at 4 months and about 71.1% among Maasai mothers said that children should be fed based on hunger cues (Table 3).

**TABLE 3:** Maasai mother's knowledge on complementary feeding (*N* = 286).

Criteria for complementary feeding knowledge	Frequency (*n*) (yes)	%
Breastfeeding		
A child should be breastfed on demand	227	79.4
Continued breastfeeding for 24 months and beyond	203	71
Complementary feeding practices		
Introduction of CF at 6 months	134	46.9
Meal frequency and energy density of complementary feeding		
Feeding a 12 months breastfed child 2 times a day	278	97.2
Feeding based on hunger cues	205	71.7
Child's porridge is made richer with butter, ghee, and oil	277	96.9
Consistency of complementary feeding		
Feeding a 6 months old child on pureed baby food	229	80.1
Children should start to eat from the family pot at 12 months	144	50.3
Hygiene during preparation and storage of complementary food		
Hands washing before preparing child's food	277	96.9
Bottle feeding is appropriate for breastfed children	207	72.4
Treatment of water for preparing food and drinks for children	59	20.6
Soap or detergent should be used	130	45.5
Feeding during illness		
Fed recovering/sick child on dilute porridge or fruit juices	270	94.4
Responsive feeding		
Helping a child eat till they are 2 years old	220	76.9
The child's primary caregiver is mother	226	79
Amount of complementary food		
1–2 tablespoonful of food are adequate meal for a 12 months child	241	84.3
More than 2 tablespoonful of food	45	15.7
Nutrient content of complementary food		
Vegetables and fruits are complimentary foods	276	96.5
Breast milk is adequate in protein even after 6 months	117	40.9

More than half (55.2%) of Maasai mothers scored highly on understanding of complementary feeding techniques and only a few (1.7%) had low knowledge, for all mothers, the average knowledge score on complementary feeding was 15.71 ± 1.9 (Table 4).

**TABLE 4:** Maasai mothers knowledge scores on complementary feeding.

Knowledge score categories	Frequency (*n*)	%
Minimal knowledge (0–10)	5	1.7
Average knowledge (11–15)	123	43
High knowledge (16–20)	158	55.2
Mean knowledge score knowledge (SD)	15.71 ± 1.9	

A chi‐square test showed a statistically significant association between Maasai mothers' knowledge on complementary feeding practices and their education level (Tables 3 and 5). This indicates that mothers are more likely to be knowledgeable on complementary feeding practices with increase in the level of education.

**TABLE 5:** Mother's sociodemographic characteristics against complementary feeding knowledge.

Characteristics	Complementary feeding knowledge (*N* = 286)			*p*
	Minimal (%)	Average (%)	High (%)	
Age (years)				
16–20	3.6	46.4	50	.398
21–30	0.6	43.5	55.9	
31–50	3.7	40.7	55.6	
Marital status				
Married	1.6	42.8	56.1	.44
Single	0	66.7	33.3	
Separated	0	71.4	28.6	
Widow	5.6	33.3	61.1	
Mother's education level				
Nonformal	4.4	44.2	51.3	.048*
Primary school	0	45.5	54.5	
Secondary school	0	36.5	63.5	
Occupation				
Housewife	1.6	42.5	55.9	.58
Petty trade/vendors	3.3	50	46.7	
Waged labor	0	0	100	

* indicate significant association at p value < 0.05.

### 3.4 Complementary feeding practices of Maasai mothers of children between the ages of 6 and 24 months

Utilizing WHO indicators for infants and young children eating patterns spanning 6–24 months, this study evaluated the Maasai mother's complementary feeding practices (WHO, 2008). The following were the warning signs: introduction of solid, semisolid, or soft foods, minimum dietary diversity, minimum meal frequency, and minimum acceptable diet.

#### 3.4.1 Solid, semisolid, or soft food introduction to children aged 6–24 months

**FIGURE 1:**
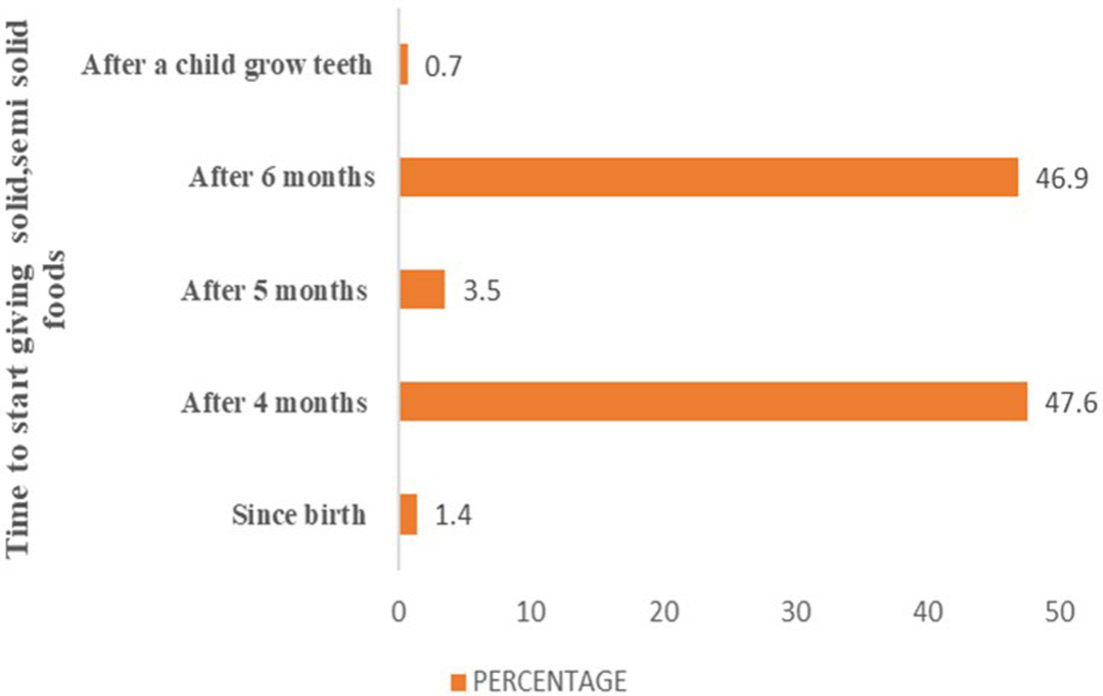
Time to start giving children solid and semisolid foods among Maasai mothers.

This represents the proportion of infants aged 6–8 months who consumed solid, semisolid, or soft foods the prior day (WHO, 2008). Based on a 24‐h dietary recall, 99.9% of the infants between the ages of 6 and 8 months got solid or semisolid foods such as rice, mashed green bananas, beans, and vegetable purée, even though 47.6% had received complementary foods prior to the age of 6 months. A smaller percentage of children (1.4%) have been fed solid, semisolid, or soft foods since birth (Figure 1).

### 3.5 Minimum dietary diversity in infants and children aged 6–24 months

The proportion of children aged between 6 and 24 months consumed food from at least five food groups the day before, including grains, roots, tubers, and plantains, pulses, meat, poultry, and eggs, as well as dairy products and other foods high in vitamin A (WHO, 2008).

**FIGURE 2:**
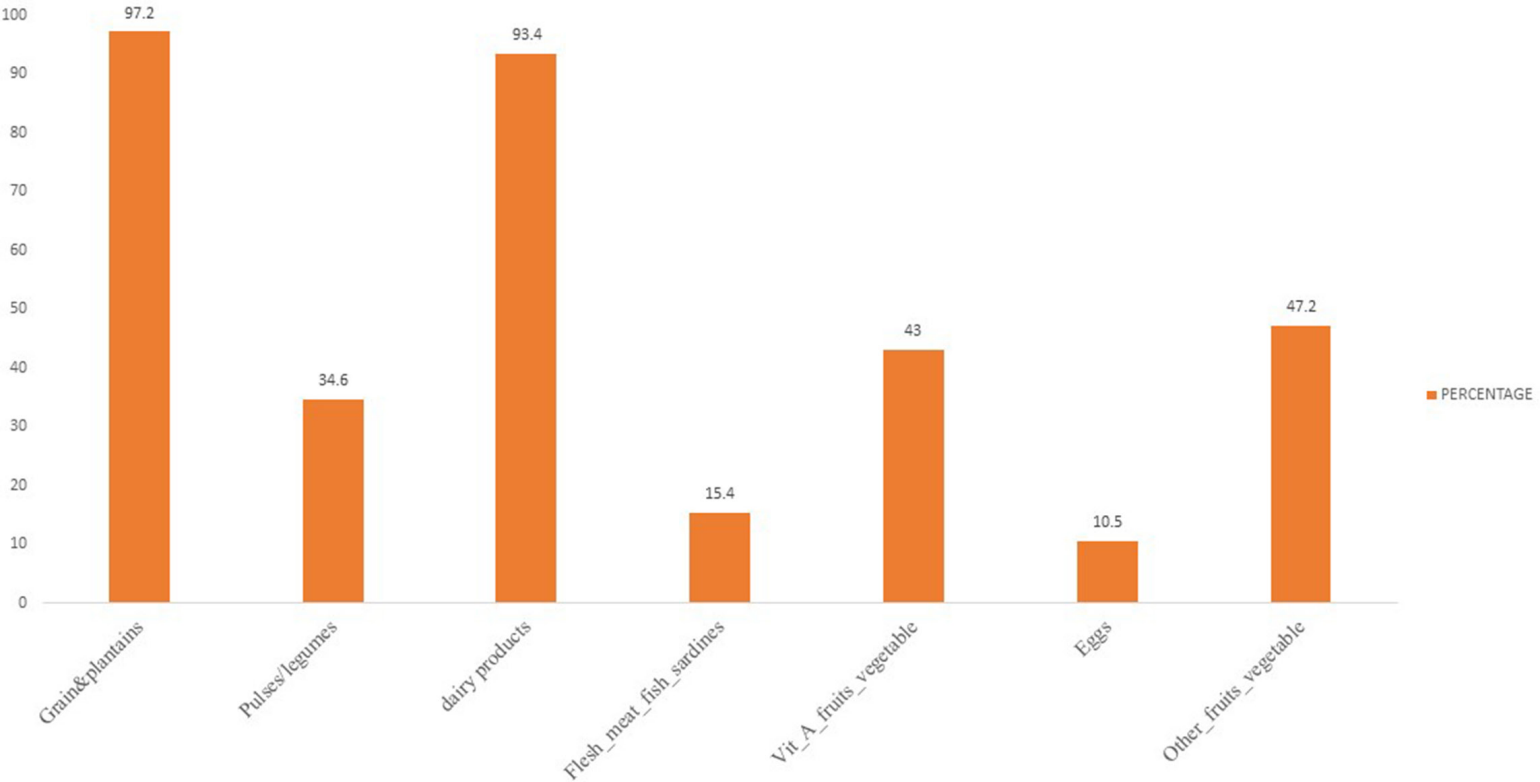
Various foods consumed by children aged 6–24 months.

Approximately 97.2% of the children had eaten meals made with plantains, tubers, and grains. Protein consumption was highest from dairy (93.4%) and lowest from pulses (34.6%) and eggs (10.5%). Fruits and vegetables high in vitamin A were consumed at a rate of 43%, while fresh meat high in iron was consumed at a rate of 15.4% and other fruits were consumed at a rate of 47.2% (Figure 2).

**FIGURE 3:**
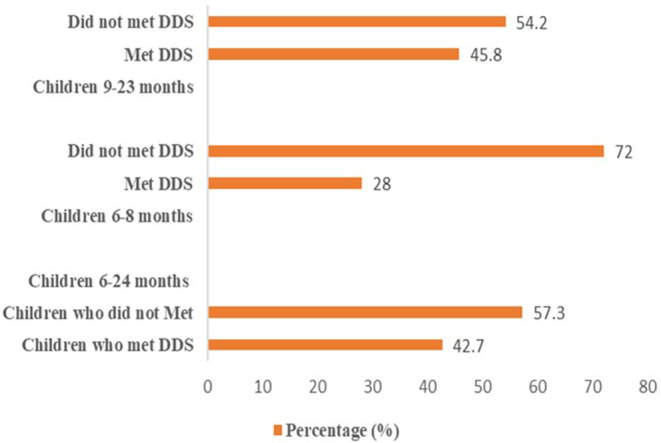
Dietary diversity score for complementary feeding among 6–24 months children.

World Health Organization (2016) defines a diversified diet for children aged 6–24 months who are fed items from at least four different food categories, whether or not they are breastfeeding. More than half (57.3%) of the children did not achieve the required minimum dietary diversity score (Figure 3).

### 3.6 Minimum meal frequency in infants and children aged 6–24 months

This is the proportion of children aged 6–24 months who ate solid, semisolid, or soft foods (including milk feeds for those who were not breastfed) at least a certain number of times or more the day before (WHO, 2008). Infants who are breastfed and are 6–8 months old should eat solid, semisolid, or soft foods twice a day, including snacks. Those who have not been breastfed should consume these foods 3 times a day, including snacks. Semisolid and/or soft foods should be eaten 3 times a day by breastfed children aged 9–24 months, and 4 times a day by nonbreastfed children aged 6–24 months, including snacks (WHO, 2008). The minimum recommended meal frequency was not met by 66.1% of the children aged 6–24 months (Table 6).

**TABLE 6:** Minimum meal frequency in infants and children aged 6–24 months.

Age of child in months	Minimum meal frequency status	%
Breastfed children (*n* = 246)		
6–8 (*N* = 50)	Attained	32
	Did not attain	68
9–24 (N = 196)	Attained	41.3
	Did not attain	58.7
Nonbreastfed children (*n* = 40)		
6–24 (*N* = 40)	Attained	0
	Did not attain	100

### 3.7 Minimum acceptable diet for infants and children aged 6–24 months (*N* = 286)

**FIGURE 4:**
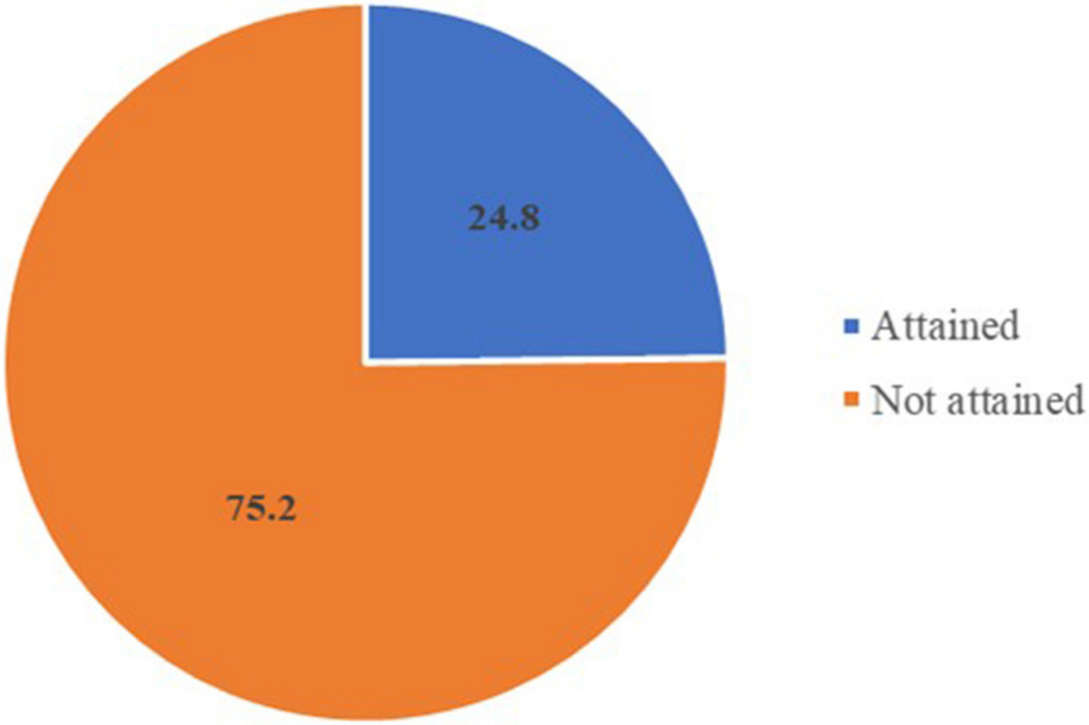
Minimum acceptable diet for infants and children aged 6–24 months.

This refers to the proportion of children aged 6–24 months who had a minimum acceptable diet the day before (WHO, 2008). The indicator is a composite of the three indicators: minimum dietary diversity, minimum meal frequency, and minimum acceptable diet. The minimal acceptable diet was not met by 75.2% of children (Figure 4).

### 3.8 Children's nutritional status in the study area (*N* = 286)

The children's nutritional status was assessed using their weight for age, length for age, and weight for height. With more boys (34.5%) than girls (25.9%) underweight, more than 80 (29.4%) children were underweight. Overall, 27.6% of the children were stunted, with girls being more likely to be stunted (30%) than boys (24.1%). When it came to wasted children, a different pattern was seen: 16.4%, with 24.1% of boys and 11.2% of girls wasted, respectively (Table 7).

**TABLE 7:** The undernutrition status between children aged 6–24 months.

	All children (*n* = 286)	Male (*N* = 116)	Female (*N* = 170)
Underweight	84 (29.4%)	40 (34.5%)	44 (25.9%)
Stunting	79 (27.6%)	28 (24.1%)	51 (30%)
Wasting	47 (16.4%)	28 (24.1%)	19 (11.2%)

### 3.9 Association between knowledge of Maasai mothers and complementary feeding

The association between complementary feeding practices and Maasai mothers' complementary feeding knowledge, classified into three categories of high, average, and low knowledge, was established. Minimum dietary diversity, minimum meal frequency, and minimum permissible diet were not significantly correlated with Maasai mothers' knowledge of complementary feeding, according to a chi‐square test (*p* = .985, .803, and .580, respectively) (Table 8).

**TABLE 8:** Association between Maasai mother's knowledge on complementary feeding and complementary feeding practices.

Complementary feeding practices	Maasai mother's knowledge on CF			
	Low knowledge (%)	Average knowledge (%)	High knowledge (%)	Chi‐square: *p*
Minimum dietary diversity				
Met MMD score	40	42.3	43	.985
Not met MMD score	60	57.7	57	
Minimum meal frequency				
Attained	20	34.1	34.2	.803
Not attained	80	65.9	65.8	
Minimum acceptable diet				
Attained	20	22	27.2	.580
Not attained	80	78	72.8	

*Note*: Significantly associated at *p* < .05.

### 3.10 Relationship between the nutritional status of children aged 6–24 months and Maasai mothers' knowledge of complementary feeding

Twenty questions selected from the WHO guideline for infant and young child feeding were used to gauge Maasai mothers' knowledge of infant and young child feeding, and their relationship to the children’ nutritional status was assessed. With respect to underweight and wasting, a chi‐square test revealed a significant association between Maasai mothers' knowledge of continuing to breastfeed a child for at least 12 months and beyond (*p* = .00 and .027, respectively). Mothers who thought that 1–2 tablespoons were sufficient for a 1‐year‐old child had a higher likelihood of having underweight children. There was no association between any other aspects on mother's knowledge of complementary feeding and the nutritional health of their children (Table 9).

**TABLE 9:** Significant relationship between the nutritional status of children ages 6–24 months and Maasai mothers' understanding of complementary feeding (*N* = 286).

Knowledge criteria on complementary feeding	Nutritional status of the child			Chi‐square; *p*
Breastfeeding for at least 2 years	**Underweight**			
		Normal	Underweight	.000*
	Agreed	79.2%	51.2%	
	Disagreed	20.8%	48.8%	
	**Wasting**			
		Normal	Wasting	.027*
	Yes	73.2%	59.6%	
	No	26.8%	40.4%	
1–2 tablespoonful of food are adequate meal for a 12 months child	**Underweight**			
		Normal	Underweight	.014*
	Yes	80.2%	94.0%	
	No	19.8%	6.0%	

* Substantial association at *p* < .05.

### 3.11 Nutritional status of the children and the Maasai mother's complementary feeding practices

Underweight, stunting, and wasting in children were explored, along with the relationship between complementary feeding strategies such as minimum dietary diversity, minimum meal frequency, and minimum acceptable diet. An underweight index of the children's nutritional status and the timing of the introduction of supplemental foods were shown to be highly associated (chi‐square test, *p* = .000). The minimal acceptable diet was significantly correlated with stunting and underweight (*p* = .027 and .001, respectively). Additionally, a chi‐square test showed a significant link (*p* = .001) between underweight and minimum dietary diversification (Table 10).

**TABLE 10:** Association between nutritional status of the children and the mother's complementary feeding practices.

Complementary feeding practices	Nutritional status of the child			Chi‐square: *p*
Minimum acceptable diet	**Stunting**			
		Normal	Stunted	.027*
	Achieved	84.5%	15.5%	
	Not achieved	68.4%	31.6%	
Minimal acceptable diet	**Underweight**			
		Normal	Underweight	.001*
	Achieved	88.7%	11.3%	
	Not achieved	64.6%	35.4%	
Minimum dietary diversity	**Underweight**			
		Normal	Stunted	.001*
	Met MDD score	83.6%	16.4%	
	Not met MDD score	64.0%	36%	
Timely introduction of complementary foods	**Underweight**			
		Normal	Underweight	.000*
	After 4 months or below	58%	42%	
	After 6 months	84.6%	15.4%	

* Strongly associated at *p* < .05.

### 3.12 The determinants of child's nutritional status

To find the nutritional status determinants, logistic regression analysis was used to further analyze the factors that appeared to be significantly associated with child nutritional status in the chi‐square test (Tables 3 and 10). In terms of complementary feeding practices, wasting and underweight were predicted by minimal acceptable diets, but underweight was predicted by minimum dietary diversity. The nutritional status of the child was also predicted by mothers' knowledge of complementary feeding, with knowledge of breastfeeding duration being a predictor of underweight (Table 11).

**TABLE 11:** Determinants of children's nutritional status.

Predictors	95% CI	Odds ratio	*p*
	**Complementary feeding practices**		
Minimum acceptable diet	Wasting 1.248–5.102	2.523	.010
Minimum acceptable diet	Underweight 1.960–9.459	4.306	.000
Minimum dietary diversity	Underweight 1.704–5.282	3.000	.000
	**Maasai mother knowledge on complementary feeding**		
Breastfeeding for at least 2 years	Underweight 2.103–6.273	3.632	.000

## 4 DISCUSSION

### 4.1 Sociodemographic characteristics of Maasai mothers and children aged 6–24 months

In this study, majority of the Maasai mothers were young with low level of education and were housewives. Similar findings were reported by Yimer (2018) in Ethiopia. Education is an important means that enable mothers/caregivers to provide appropriate care for their children, hence an important factor for children growth and development (Levin et al., 2000). Low levels of education may have contributed to most of the Maasai mothers being housewives. Mothers reported that household income was inadequate to provide food and other basic needs. Priority of the household resources was placed on livestock first and then food and other health‐related needs. This may have contributed to the majority of the household's failure to attain minimum acceptable diet for the children.

### 4.2 Complementary feeding practices in infants and children between the ages of 6 and 24 months

#### 4.2.1 Breastfeeding practices

This study found that almost all of the children had been breastfed and this compares to the Tanzania figures as reported in Tanzania Demographic Health Survey (TDHS, 2015–2016). This was expected due to the fact that culturally Tanzanian women especially from rural areas breastfeed their children. Breastfeeding throughout complementary feeding for infants and young children aged 6–24 months contributes significantly to the overall nutrient intake and fill the energy needs and also remains an important source of vitamins A, C, and essential fatty acids (Mukuria et al., 2006). More than three quarter of Maasai mother reported to practices continued breastfeeding up to 24 months of age. This is important as it could prevent half of all deaths associated with infectious diseases between 6 and 24 months of age (Sankar et al., 2015).

#### 4.2.2 Timely introduction to solids, semisolids, and soft foods

This study found that Maasai mothers did not introduce complementary foods timely; about 47.6% of mothers claimed to introduce weaning at age less than 6 months. This result was similar to the findings of a study conducted in Marsabit county, Kenya, where only 50% of infants were properly introduced to supplemental foods at 6 months (Mutuku et al., 2020). If complementary foods are not introduced timely, sooner or after a child reach 6–8 months of age, an infant's health and growth will be at higher risk of being stunted or underweight (WHO, 2001). From the study, more than half (53.1%) of Maasai mothers were not aware about the ideal window for weaning, despite the fact that the timing of the introduction of adequate and safe complementary foods is crucial for improved child nutritional status and well‐being, particularly during times of increased nutritional needs (WHO, 2003). In developing countries, it is typical to introduce complementary foods earlier than the advised age of 6 months, based on research by Shumey et al. 2013; Safari et al. (2013) and Burns et al. (2016).

#### 4.2.3 Minimum dietary diversity

Although the intake of high‐quality diversified diets is thought to ensure adequate intake of essential nutrients and hence promote good health and nutrition (Chessa, 2003; Lutter & Rivera, 2003), the study showed that only 42.7% of children had eaten diversified diets at least from four food groups. This was higher as compared to the Tanzania national figures (24%) as reported in Tanzania Demographic Health Survey (TDHS, 2015–2016). A poor diversified diet can increase the risk of micronutrient deficiencies, which may have detrimental impact on children's physical and cognitive wellness (Prado & Dewey, 2014). Moreover, majority (97.2%) of the children had eaten cereal‐based foods like rice, potatoes, porridge, cassava, mashed green bananas, and maize stiff porridge. These were the complementary food choices frequently consumed due to the fact that maize is the primary food source in the most of Tanzanian households (Mollay et al., 2021). This can be linked to the fact that being a low‐income and semiarid area as Naitolia, Maasai mothers would prefer cheap foods which are usually grain, roots, tubers, and their products and the fact that cereals form the staple food of most communities in Tanzania. Nevertheless, maize porridge was most taken by almost all of the children in this study as the common complementary food. Similarly, a study conducted in western Kenya reported high consumption of porridge (Mbagaya, 2009).

The intake of foods high in vitamin A was inadequate and diminished with age. This was similar to the study conducted in Kenya by Chege et al. (2015). Vitamin A deficiency is one among nutritional deficiencies of supreme public health importance in the world recently. About one third of children in developing countries are affected to some extent by vitamin A deficiency, which weakens their growth, vision, and immune system, and in extremes cases leads to blindness and death (Gretel & Margaret, 2011).

The consumption of iron‐rich foods (flesh meat, sardines) was low. Despite the fact that Maasai people are pastoral community, food of animal's origin was not consumed by the majority of the households. The consumption of protein was highest from dairy products such as a milk and ghee, in agreement with the study findings by Lawson (2014) in northern Tanzania. Even among the pastoralist Maasai group, who are believed to consume meat more frequently than crop farmers, other animal food sources (meat, fish, and poultry items) were less consumed than milk due to food taboos. In addition, studies conducted in Ethiopia and Kenya found the same results of less frequent meat intake in pastoralist communities (Chege et al., 2015; Masresha et al., 2013). Other studies in pastoral communities have showed similar findings, for example, in Marsabit county and Turkana, Kenya revealed low minimum dietary diversity of 15.5% and 19.5%, correspondingly (UNICEF, 2017, 2018). The low minimum dietary diversity could be due food insecurity which results to poor food availability and accessibility (Mutuku et al., 2020).

#### 4.2.4 Minimum meal frequency

In this study, most of the breastfed children did not attain minimum meal frequency same as their matching part, the nonbreastfed children. This finding was quite similar with that of the study conducted in Kenya by Kipruto (2013). Frequent meals, 2–3 meals and 3–4 snacks per day are the least recommended meal frequency for healthy breastfed infants between the ages of 6 and 8 months and 9 and 24 months, with an additional one or two snacks between meals for children between the ages of 6 and 24 months who are not breastfed (WHO, 2008). In this study, majority of Maasai mothers fed their children twice a day, regardless of their age groups and breastfeeding status, while more than half (71.1%) of Maasai mothers fed their children based on hunger cues which could be the influence on their feeding frequencies. This was in agreement with the study done in Jigjiga, Ethiopia, where the population relies on pastoralism as a means of subsistence (Yimer, 2018). According to other studies conducted in Tanzania and Kenya, this may be intensified in part by the fact that children are typically fed as part of the family mealtime pattern in most households (Chege et al., 2015; Nyaruhucha et al., 2006).

#### 4.2.5 Minimum acceptable diet

In this study, three quarter of the children did not attain minimum acceptable diet, indicating low‐quality diet consumed by most of the children. The findings are similar with those of Marsabit County (UNICEF, 2018). Infant and young children should be fed a minimum acceptable diet to ensure appropriate growth and development. Without adequate diversity and meal frequency, infants and young children are vulnerable to undernutrition, especially stunting and micronutrient deficiencies (WHO, 2006). This finding can be linked to giving children diets low in variety and at incorrect meal intervals, likely as a result of localized food insecurity and poor knowledge on complementary feeding. Infants who get inadequate complementary feeding may experience delayed growth, increased risk of undernutrition, and anemia (Huo et al., 2021).

### 4.3 Knowledge on complementary feeding practices among Maasai mothers

Generally, Maasai mothers had average knowledge on complementary feeding practices. This finding was similar with study carried out in Marsabit County, Kenya (Mutuku et al., 2020). On contrary, a high knowledge on complementary feeding practices among mothers was found in the study conducted in Ethiopia (Yimer, 2018). Knowledge on complementary feeding is important resource for enhancing young children's health and well‐being (WHO, 2016). Almost all mothers were aware about the duration for exclusive breastfeeding and duration of breastfeeding. UNICEF and World Health Organization recommendations emphasize that children should be exclusively breastfed for 6 months and breastfed continues for 2 years and beyond (WHO, 2016). Maasai mother's knowledge on feeding frequency was high as majority of mothers reported that children should be fed based on hunger cues. Appropriate complementary feeding practices includes adequate meal frequency depending on whether the child is breastfeeding (WHO, 2016). Majority of the Maasai mothers were aware of the importance enriching foods and providing a diverse diet to their children. This is an important aspect in the infant and young children feeding practices as it increases the nutrient density. Furthermore, Maasai mothers did the appropriate flour for making child's porridge as recommended by the World Health Organization on feeding infants and young child (WHO, 2016), therefore inappropriate flour mixes of cereal and legume were used to prepare porridge. Also, during the focus group discussion, Maasai mothers reported that the most appropriate flour for preparing child's porridge was a mixture of maize, millet sorghum, and cassava. The mixture of cereals and legumes flour contains phytates which bind iron, hence not appropriate for children (UNICEF, 2021). Moreover, legumes take longer to cook than cereals and if not precooked can led to gastrointestinal disturbance in children.

Normally, appetite may be reduced when a child is sick; continued consumption of complementary foods is recommended to maintain nutrient intake and facilitate recovery (Brown, 2001). This was quite the opposite in this study as Maasai mothers reported that children who are sick and those recovering should be fed on dilute porridge and fruit juices only. This would be probably among the cause of high undernutrition especially underweight and wasting among children. During recovering, the child requires more nutrients intake to counterbalance the nutrient losses during the illness. More food is required until the child has reacquired the weight lost and is growing appropriately (WHO, 2001).

### 4.4 Nutritional status among Maasai children

Underweight and wasting rates in this study were higher than the Tanzania national figures in children under 5 years. This was possible because the study considered children aged 6–24 months. Stunting was quite lower than the national figure (TDHS 2015–2016) as stunting rate increases with child age. A study conducted in Simanjiro by Nyaruhucha et al. (2006) found that the prevalence of malnutrition among Maasai children rose with the children's ages. The increased frequency of underweight and stunting in pastoralist communities may be related to a number of social, health, and economic issues that other research have taken into consideration for pastoralist groups (Downie, 2011). A study conducted in Tanzania by Lawson (2014) revealed that, in comparison to nearby ethnic groups, the Maasai were severely disadvantaged. This study determined only the influence of mother's knowledge on complementary feeding practices and hence nutritional status. Therefore, it is impossible to explain what factors could have caused the high prevalence of undernutrition in the study population.

### 4.5 Relationship between Maasai mother's understanding of complementary feeding and the nutritional status of infants and children aged 6–24 months

For mothers or caregivers to improve their feeding practices for infants and young children, appropriate information about complementary feeding is crucial (WHO, 2003). The nutritional status of the Maasai children aged 6–24 months was shown to be strongly associated with the knowledge of the mothers in this study. However, factors like culture beliefs, mother's perception, and food availability have influences on complementary feeding practices, hence, the nutritional status of the child (Sellen, 2001). Mothers who knew the right time to introduce supplemental foods were more likely to have children with good nutritional status. Nevertheless, mothers who knew the time period for breastfeeding a child (12 months and beyond), their children were less likely have wasting and underweight. Similar results were found by the study done in Marsabit county by Mutuku et al. (2020). Although Maasai mothers were aware of complementary feeding practices, the study's findings indicated that they did not use these techniques. According to the finding in the study by Mutuku et al. (2020) revealed that factors besides knowledge affected supplementary feeding behaviors. Additionally, this study's findings indicated a link between children's nutritional status and achieving minimum dietary diversity, meal frequency, and acceptable diets. This means that the child's nutritional condition was influenced by the mother's complementary feeding practices. Similar to this, a study by Okwori et al. (2011) and Tessema et al. (2013) found a substantial relationship between nutritional status and feeding patterns.

## 5 CONCLUSION AND RECOMMENDATIONS

The study population comprised young housewives' mothers with low level of education. Mother's knowledge on complementary feeding was somewhat appropriate; gaps were identified in feeding the sick children and on preparation of the correct mixture of porridge flour. Maasai mothers' complementary feeding practices were unsatisfactory; in terms of introduction of complementary foods, dietary diversity, and meal frequency for children. The prevalence of undernutrition among infants aged 6 and 24 months was high, and was associated with complementary feeding practices and complementary feeding knowledge of mothers. The nutritional status of children in the Monduli District should be improved through strategies that go beyond educating parents about adequate complementary feeding practices as in semiarid areas like Monduli District, household food insecurity being among the major contributor to inadequate infants and young children feeding practices, efforts should be done to address the problem.

### 5.1 Strengths and limitations

This is the one of the few studies to assess mothers/caregiver knowledge on complementary feeding practices and nutritional status of the children aged 6–24 months among pastoral community in Tanzania. The study was conducted at household level which offers observations on different feeding practices. Thus, findings will provide baseline information to policymaker and public health practitioners to implement evidence‐based interventions within country to improve infants and young children feeding practices through emphasis on the knowledge and practices among mothers or caregivers. The limitation of this study includes the use of cross‐sectional study design which influences the answers and practices by the respondents. A longitudinal study would give a clear picture of what is going on in the area. The study was conducted only in part of Monduli District among Maasai mothers, therefore, findings cannot be generalized among Maasai women across the country.

## AUTHOR CONTRIBUTIONS

**Jovin Binamungu:** Conceptualization (lead); data curation (equal); investigation (equal); methodology (equal); project administration (equal); writing – original draft (equal); writing – review and editing (equal). **Sharadhuli I. Kimera:** Data curation (supporting); funding acquisition (supporting); methodology (equal); resources (supporting); supervision (equal); validation (equal). **Beatha Mkojera:** Conceptualization (supporting); data curation (supporting); supervision (equal); writing – review and editing (supporting).

## CONFLICT OF INTEREST STATEMENT

The author declares no conflict interest.

## Data Availability

The data that support the finding of this study are available on request from the corresponding author.
